# Producer perceptions on the impacts of the withdrawal of zinc oxide on the health and welfare of weaned pigs

**DOI:** 10.3389/fvets.2026.1717403

**Published:** 2026-04-20

**Authors:** Abel B. Ekiri, Sam Beechener, Ilias Kyriazakis, Nick Wheelhouse, Andrew G. Bease, Nicky Craig, Sadie Douglas, Sophie Prentice, Samuel A. M. Connelly, Manal AbuOun, Muna F. Anjum, Mark P. Stevens, Roberto M. La Ragione, Deborah V. Hoyle

**Affiliations:** 1 Department of Comparative Biomedical Sciences, School of Veterinary Medicine, University of Surrey, Guildford, United Kingdom; 2 Scotland’s Rural College (SRUC), Edinburgh, United Kingdom; 3 Institute for Global Food Security, Queen’s University, Belfast, United Kingdom; 4 School of Applied Sciences, Edinburgh Napier University, Edinburgh, United Kingdom; 5 The Roslin Institute, Royal (Dick) School of Veterinary Studies, Easter Bush Campus, University of Edinburgh, Edinburgh, United Kingdom; 6 AB Neo/AB Agri Limited, Peterborough, United Kingdom; 7 Bacteriology Department, Animal and Plant Health Agency, Addlestone, United Kingdom; 8 Discipline of Microbes, Infection, and Immunity, School of Biosciences, University of Surrey, Guildford, United Kingdom

**Keywords:** antimicrobial resistance, health, mixed methods, pig, post-weaning diarrhoea, welfare, zinc oxide

## Abstract

**Background:**

Zinc oxide (ZnO) added to pig feed at prophylactic levels has been used to reduce post-weaning diarrhoea (PWD) and enhance performance. In response to concerns about antimicrobial resistance (AMR) and environmental contamination, supplementation with ZnO was prohibited in the European Union from June 2022, and in the United Kingdom from June 2024.

**Objective and method:**

We investigated the impacts of the withdrawal of ZnO on the health and welfare of weaned pigs as perceived by pig producers, and the management and husbandry factors associated with PWD in the United Kingdom. A mixed methods approach combined qualitative interviews with producers preparing to withdraw ZnO from their herds and a quantitative survey open to any producer. Findings were integrated using the transtheoretical model (TTM) of change as a guiding framework.

**Results:**

Eight qualitative interviews and 14 quantitative surveys with producers were completed. Responses to the qualitative interviews were all from indoor, breeder-finisher herds while the quantitative survey included indoor and outdoor production systems and a mix of breeder-finisher herds and nursery units. In the qualitative interviews, producers described ZnO as affordable and effective. Concerns were expressed that its withdrawal risked triggering a sectoral increase in antimicrobial usage, jeopardising reductions achieved to date. The quantitative survey revealed that almost all respondents (12/14, 85%) had experienced PWD in their piglets in the last year. At the time of responding, 57% of respondents (8/14) reported weaner pigs were not fed a diet containing ZnO during the post-weaning period whereas 36% (5/14) were still feeding a diet containing ZnO. Following the withdrawal of ZnO in June 2024, most respondents (8/14, 57%) anticipated supplementing weaner diets with an alternative to ZnO before and after weaning.

**Conclusion:**

While recognising the limitations of a small sample size, the study contributes to our understanding of producer perceptions as they prepared for the withdrawal of ZnO. Producer concerns that PWD would be less effectively controlled in their herds and risked triggering an increase in antimicrobial usage were highlighted. Further work is required to better understand the type and effectiveness of alternatives to ZnO on pig health and welfare.

## Introduction

Post-weaning diarrhoea (PWD) is a multifactorial condition occurring in the first 14 days post-weaning, at a time when piglets are vulnerable to infection. The condition is associated with diarrhoea (1, 2) and poor weight gain of piglets (3, 4) and causes considerable economic loss to the pig industry due to the costs related to treatment, slower growth, and increased mortality (5). The primary aetiological agent of PWD is enterotoxigenic *Escherichia coli* (ETEC), with—F4 fimbriae positive isolates (previously K88) currently predominant in Europe (6). The role of other pathogens in PWD such as Rotavirus is also recognised (7).

In the European Union and the United Kingdom, prior to its withdrawal, zinc oxide (ZnO) was used at pharmacological levels of 2,500 ppm as a dietary additive to manage PWD by some producers in the pig industry. Supplementation with ZnO reduces the incidence of PWD in weaner piglets (8, 9) and improves production measures such as growth performance (10, 11), digestion (9), and feed intake (12). Although ZnO has beneficial effects, ZnO usage may have negative effects on the environment, through contamination of soil with zinc excreted in animal slurry and manure waste (13). The accumulation of ZnO in the environment has been linked to toxicity in sediment organisms and aquatic species, with negative effects on the ecological balance (14, 15). Diet supplementation with ZnO is thought to contribute to the acquisition and spread of AMR: high level ZnO supplementation has been associated with an increase in the isolation of multi-drug-resistant *Escherichia coli* from gut content and pig faeces (16–18) and of methicillin resistant *Staphylococcus aureus* from nasal swabs (19).

The negative effects of ZnO on the environment and AMR raise important livestock, food safety and public health concerns. In response to these issues, the use of ZnO was prohibited in the EU from June 2022 (20), and in the United Kingdom from June 2024, requiring producers to apply alternative strategies to manage PWD in the absence of dietary ZnO, such as management practices, including weaner nutrition (21). Given withdrawal was signalled from 2017, pig producers in the United Kingdom were at different stages in terms of considering and implementing alternative strategies by the time the ban came into effect. A study of Danish pig herds (22) found that while producer experience of phasing out ZnO was mostly positive, in some cases considerable challenges were encountered, including diarrhoea and oedema disease and increased usage of antibiotics, for example including the metaphylactic treatment of entire batches rather than individual animals. In the United Kingdom, there is limited data on producer perceptions of the impacts of the withdrawal of ZnO on the health and welfare of weaning pigs.

The aims of the current study were to improve our understanding of the perceived challenges and impacts experienced by producers in relationship to management of PWD, and the factors that may mitigate the impact of the withdrawal of ZnO on the health and welfare of weaned pigs. The above aims were achieved through the following objectives (1) investigating attitudes among pig producers as the United Kingdom sector prepared for the withdrawal of ZnO; and (2) identifying management and husbandry factors associated with PWD. We applied the transtheoretical model (TTM) of change (23) as a framework to assist in understanding changes in producer attitudes and behaviours during the transition, and perceptions of the impact of withdrawal of ZnO on the health and welfare of weaning pigs.

## Methods

This work was part of a larger, longitudinal study, running between 2022–2025, examining the impact of ZnO withdrawal on PWD and AMR carriage during the first 12 months following ZnO withdrawal in commercial United Kingdom pig herds (*n* = 29).

### Study population and design

**Figure 1:**
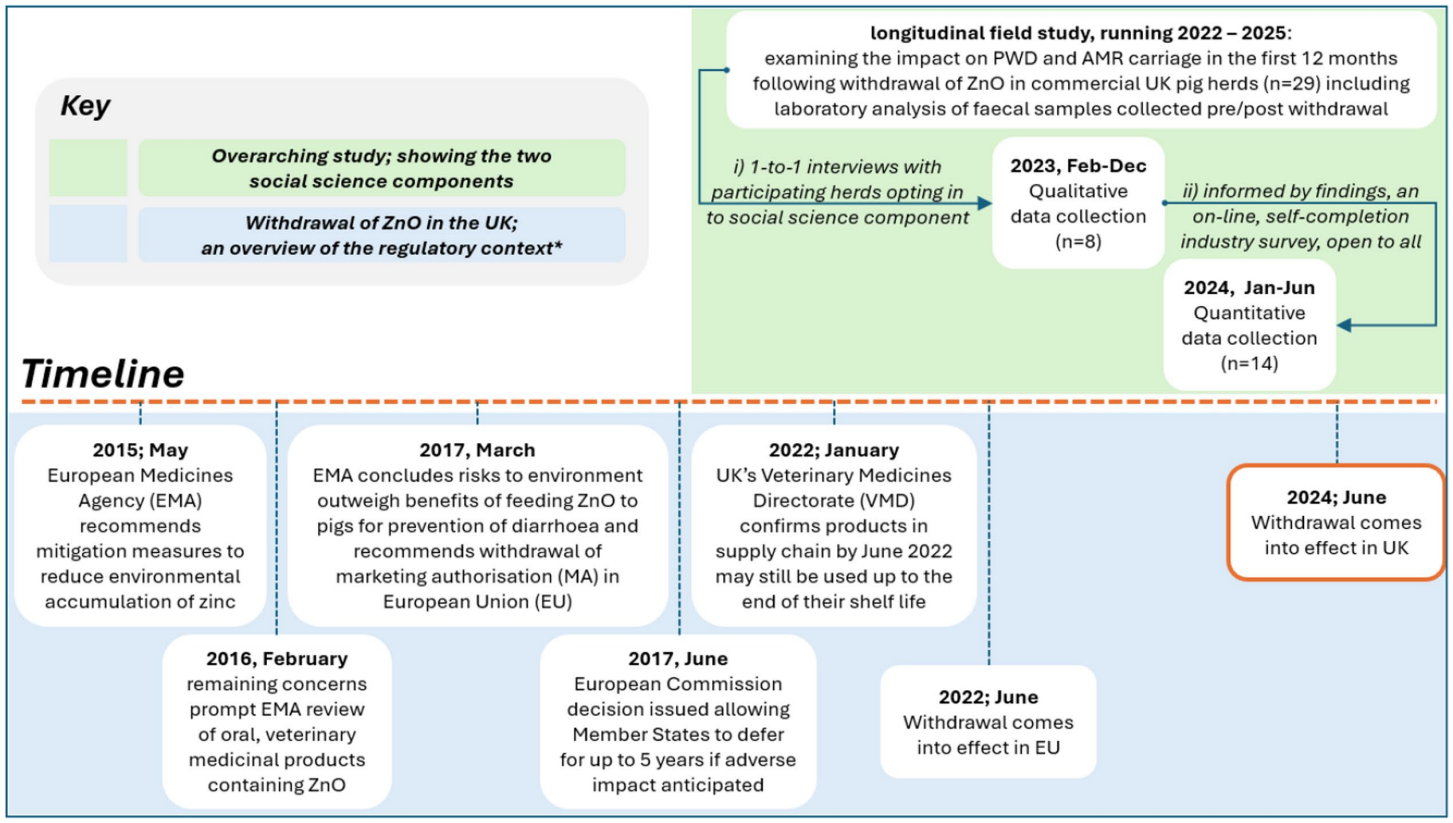
An overview of the research approach in the context of the timeline to withdrawal of zinc oxide (ZnO) in the United Kingdom. *Source: Zinc oxide—referral|European Medicines Agency (EMA) Withdrawal of marketing authorisations of veterinary medicines containing zinc oxide—GOV.UK (accessed December 04, 2024).

The study targeted pig producers from the United Kingdom. The work reported here comprises two components: qualitative interviews and quantitative epidemiological survey. An exploratory mixed methods approach (24) was applied with findings from qualitative interviews informing the design of a quantitative survey. A visual summary of the timeline for the qualitative interviews and quantitative surveys relative to the United Kingdom ZnO withdrawal date of June 2024 is shown below (Figure 1). Qualitative interviews were conducted with a subset of producers recruited to the above longitudinal study. For the quantitative epidemiological survey, an online, anonymous survey was conducted, and disseminated to United Kingdom producers through specialist press, producer groups and social media sites. Details of participant enrolment, and data collection and analysis for the qualitative and quantitative components are set out below.

### Qualitative interviews: attitudes and perceptions

#### Participant enrolment

Participants were recruited to the qualitative study from a cohort of 29 United Kingdom herds initially enrolled to a longitudinal observational study. The 29 recruited herds in this study (median 650 sows, range 100–1,100) fall within the scope of medium to very large commercial United Kingdom enterprises, representative of the upper tier of United Kingdom commercial production holdings. In total, aggregated sow numbers across the 29 herds at point of enrolment to the study numbered approximate 16,690 breeding females. Based on the 2025 United Kingdom census data, the total United Kingdom breeding herd numbered 316,000 females, thus herds recruited to the longitudinal study represent approximately 5% of the United Kingdom breeding herd. On recruitment to the longitudinal study, producers were invited to opt in to a 1:1 interview on attitudes and perceptions.

#### Data collection and analysis

An interview guide was developed, consisting of a mix of open and closed questions. Open questions explored the impacts experienced and anticipated at farm level as the sector approached the ZnO withdrawal date; closed questions used a five-point scale, (disagree strongly, disagree, unsure, agree, agree strongly) to gauge response to given statements. Interview questions were pre-tested with industry contacts and revised following feedback. The interview guide (Supplementary File 1), participant information sheet and consent form were uploaded to the JISC online survey platform, and access shared with participants as they opted-in to the social science component over the period February to December 2023. By providing the participants with flexibility to select a one-to-one interview or to self-complete the form, we sought to minimise the burden of engagement. All participants chose to be interviewed, appointments were made, informed consent was obtained and conversations, lasting approximately 30–40 minutes were conducted via digital communication. Responses were captured by the interviewer on a form uploaded to the JISC online survey platform. These records provided the basis for qualitative descriptive analysis. Data were arranged, analysed and interpreted in sequence (25) and according to the chronology of integrated production systems, moving from farrowing and pre-weaning through weaning and into post-weaning stages. Emerging findings from the qualitative interviews were used to inform development of the quantitative epidemiological survey (described below).

### Quantitative survey: epidemiological considerations

#### Participant enrolment

The epidemiological survey was open to all pig producers across the United Kingdom, reflecting the main farming systems, including standard indoor bred, higher welfare indoor bred, outdoor bred, and outdoor raised.

#### Data collection and analysis

Guided by responses to the qualitative interviews (above), a detailed epidemiological survey (Supplementary File 2) was developed. The survey consisted of questions about the farm and herd characteristics, pig husbandry, management, and health. The online survey was prefaced by a participant information sheet and consent form and hosted through the JISC online survey platform, all respondents were required to confirm reading of these documents and provide informed consent, before proceeding to complete the survey. Participants that declined consent were unable to proceed to the survey. The survey was pre-tested with six respondents (including four veterinarians, one social scientist, and one pig producer) and it was revised following feedback. A survey link and QR code were created and shared with pig producers through industry contacts, via their newsletters and social media contacts. Participants followed the survey link and self-completed the electronic survey.

The survey was launched in January 2024 and closed in June 2024 when the withdrawal of ZnO came into effect. Descriptive statistics (frequency distributions) were undertaken using Microsoft Excel to analyse and summarise responses to the survey. Fisher’s exact test was performed in SPSS (IBM SPSS version 29.0) to explore potential associations between respondents reporting PWD in weaned piglets and select variables of interest.

### Integration of qualitative and quantitative findings

Initially we report our qualitative and quantitative findings separately and later draw them together according to the five stages (pre-contemplation, contemplation, preparation, action, and maintenance) described by the transtheoretical model (TTM) of change (23, 26). The TTM integrates processes and principles of change as understood from different theoretical perspectives and traditions. Crucially, it understands behaviour change as staged over time (27), making it well suited to the extended lead-in to the withdrawal of ZnO in the United Kingdom. In the current study, the TTM is used as a framework to structure discussion of change over time in response to the regulatory withdrawal of ZnO and informed by both our qualitative and quantitative research findings. Rather than designing a behavioural study and delineating the stages of change, we applied the TTM to assist with interpretation of the different stages at the time of data collection. Qualitative and quantitative data were not combined as such, but our interpretation was integrated through the lens of the TTM. As illustrated in Figure 1, the regulatory withdrawal of ZnO was subject to an extended lead-in and the topics investigated in the qualitative interviews reflected wider discussions taking place across the industry and reported in the trade press at that time.

## Results

### Qualitative interviews: attitudes and perceptions

Eight out of the 29 producers opted-in and completed the qualitative interviews. At the time of being interviewed, two participants were in the early stages of withdrawing ZnO while six were still using ZnO and to assist with analysis they are described, respectively, as “action” and “pre-action” (28).

#### ZnO usage status

All participants interviewed had used ZnO in weaner diets. One respondent commented that ZnO “was popular for a reason, it was effective and affordable, especially in light of recent pressures on the sector” and this perception was widely shared among respondents.

Most respondents (*n* = 7/8, including both “action” respondents) agreed/agreed strongly with the statement “at present, PWD is being well-controlled in my herd.” One respondent (still using ZnO) was “unsure”, reflecting their experience of trialling ZnO-free feeding successfully on one site but less successfully on another. For them, this highlighted the challenge of finding site-specific solutions in the absence of a like-for-like alternative.

Although respondents believed that PWD was being well-controlled in their herds, five of the six respondents still using ZnO agreed with the statement “the forthcoming withdrawal of ZnO will make it more difficult to prevent and control PWD on my unit.” One respondent was “unsure” and several respondents expressed a hope that the decision to withdraw ZnO may, in time, be reversed and its targeted use allowed in the future.

Despite concerns about the impact of the withdrawal of ZnO, replies were evenly divided as to whether “more information is needed about the implications for producers of the forthcoming withdrawal of ZnO.” On the one hand, respondents tending to agree sought more information about potential alternatives; on the other hand, those tending to disagree believed there was already sufficient information on the topic for farmers. The main sources of information about piglet health at weaning were the veterinarian (mentioned by all respondents) and nutritionist (5 mentions). Other information sources included: feed provider (4 mentions), discussion groups (3), other farmers/peers (3), trade press (2) and farm consultants (1).

#### Understandings on potential impacts of the withdrawal of ZnO

Understandings about the rationale for the withdrawal of ZnO varied. For four respondents, the withdrawal of ZnO was linked to EU regulations and concerns about contamination of soils with zinc. However, they were not convinced this was a problem in the United Kingdom. Two respondents understood ZnO was being withdrawn in response to concerns about its over-usage across the sector. While one respondent suspected it had likely been over-used in their own herd; the other was sceptical given that its use is restricted to a specific target group (weaners) for a defined, two-week period. Associations between ZnO and AMR were mentioned by two respondents as a factor contributing to ZnO withdrawal.

Asked to what extent they agreed or disagreed with the statement “the forthcoming withdrawal of ZnO risks triggering an increase in the use of antimicrobials across the sector” most respondents either agreed (*n* = 3/8) or agreed strongly (*n* = 2/8). They expressed a shared concern that despite the sector’s determination to minimise the use of antibiotics, the withdrawal of ZnO, not just routinely but also as a valued “back-stop,” risked triggering an increase in antimicrobial use. Two respondents were not sure, and one respondent disagreed in the belief that the sector’s commitment to reduce antibiotic usage was sufficiently robust to safeguard against any increase.

Respondents were asked to describe how the withdrawal of ZnO was impacting or anticipated to impact on their herd’s health and on-farm activities. The two “action” respondents (early stages of ZnO withdrawal at the time of the interview) described having moved weaned pigs onto lower protein diets to mitigate for the withdrawal of ZnO. One respondent reflected that the withdrawal of ZnO initially left them feeling exposed, in their own words “like a soldier going into battle without a gun!”. The experience of both herds, however, was positive.

Among the six “pre-action” respondents (yet to withdraw ZnO), two were actively trialling ZnO-free feeding with mixed results. Both were managing pigs across multiple sites, one reported having successfully introduced a new feeding regime on one of two units but was yet to find a solution that worked on the second unit, with ZnO remaining as backstop at the time. The second described “banking the easy wins” by removing ZnO from their highest-health units but expressed concern about the challenge of withdrawing ZnO across the remainder of their herds while maintaining growth rates and performance. For a third respondent, problems had been encountered after having moved to ZnO-free diets, they had reverted to ZnO and expressed regret at its blanket withdrawal and the loss of ZnO as a “safety net.” Another two respondents suspected the withdrawal of ZnO would leave them with an increased reliance, albeit reluctantly, on antibiotics. One went on to describe having been warned of the need to adjust their expectations of what a “well-weaned pig” looks like as they learn to accept looser faeces and dirtier stock. They went on to express concerns about how this image “would look” to assurance inspectors and the wider public given increasing scrutiny on production systems. The sixth of six respondents yet to withdraw ZnO spoke of an increased focus on piglet health at pre-weaning with a view to producing more “robust weaners,” resilient to the challenges of weaning.

#### Farrowing and pre-weaning strategies to reduce the risk of PWD

Farrowing and pre-weaning strategies to reduce the risk of PWD: Most respondents (*n* = 7) agreed (*n* = 1) or agreed strongly (*n* = 6) with the statement “weaning is challenging for piglets” and agreed strongly (*n* = 7) that “reducing the challenges of weaning helps to reduce the risks of PWD”. One respondent was “unsure”, reflecting their belief that given careful management, weaning need not be challenging. Indeed, close attention to management was central to the various strategies in place to prepare pigs for the weaning process and, in the words of one respondent, “to set them up for lifetime performance.”

Respondents saw sow management, and nutrition in particular, as an integral part of this process. The focus in late gestation was to ensure sows are in good condition at farrowing: (i) target birthweights are achieved; and (ii) a plentiful supply of quality colostrum is available. In helping newborn pigs make the best possible start, respondents recognised the value of having sufficient staff with the necessary experience in the farrowing house. Their activities were supported by attention to housing, nutrition and health. For housing, the importance of ensuring good hygiene and warmth for piglets was highlighted. Although various feeding strategies were described and some respondents spoke of creep feeding as part of the “art” of stockkeeping, they shared a common aim of encouraging the intake of solid feeds ahead of weaning. For health management, most (*n* = 6/8) respondents vaccinated piglets 3–5 days pre-weaning to reduce handling on weaning day. Two respondents, however, vaccinated on weaning day to avoid repeat handling. As one respondent commented, “no two pig units are the same” and what works on one, may not work on another.

While the routines themselves differed, well-established routines were central to reducing the stress of weaning day including, for example: sorting into evenly matched groups; attending to the post-weaning housing hygiene (washing, disinfecting and drying); temperature and air quality; and ensuring ready access to clean, fresh and familiar feed and water.

#### Post-weaning strategies to reduce the risk of PWD

**Figure 2:**
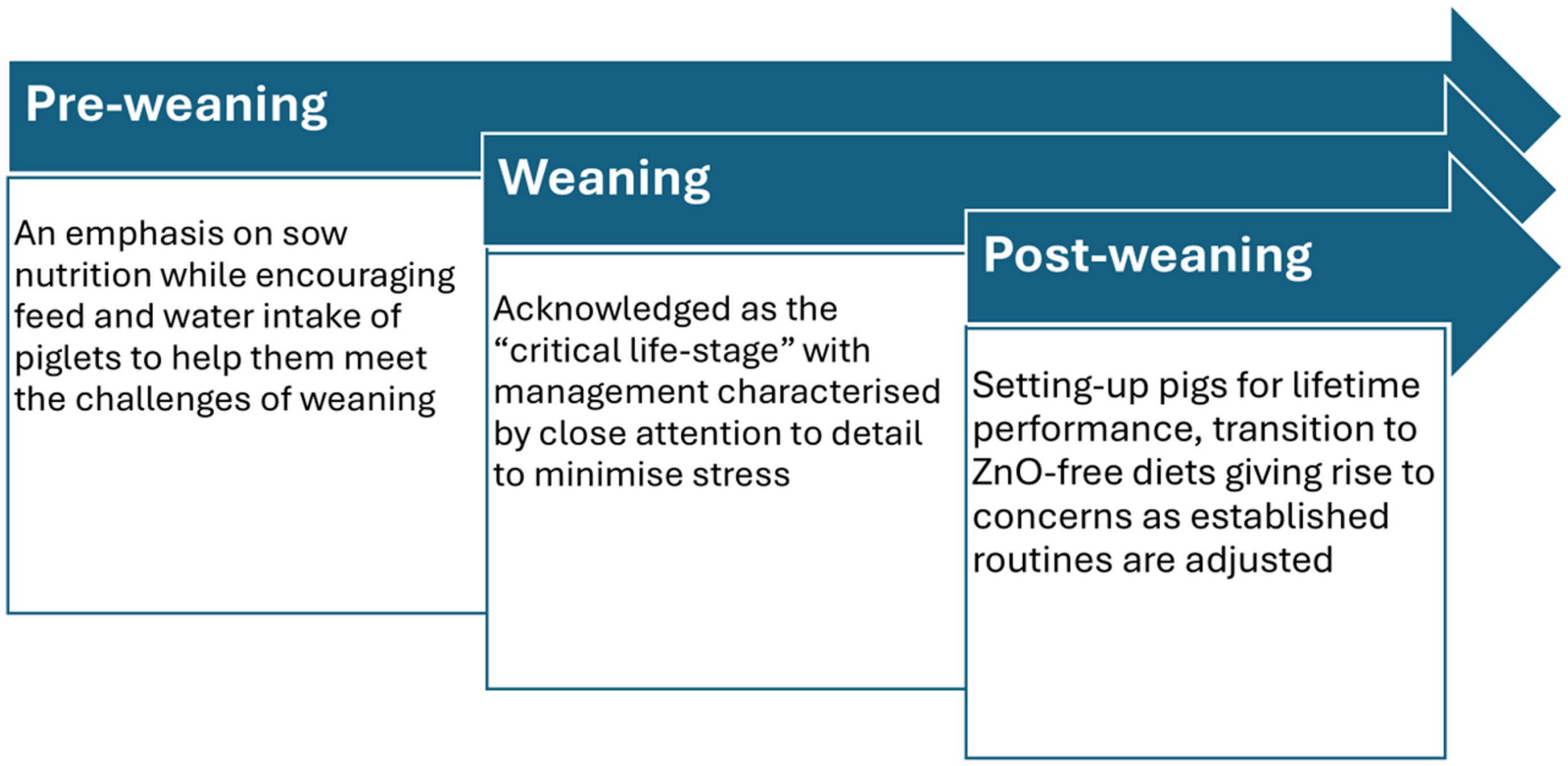
An overview of management strategies as described by participants (*n* = 8) in the context of weaning and the forthcoming withdrawal of ZnO.

The attention to detail that characterised the pre-weaning and weaning stages continued over the 2 weeks immediately after weaning. Indeed, as illustrated in Figure 2, below, the focus of management strategies is on supporting piglets over the weaning process.

As one respondent observed, “it’s about getting them through those first 2 weeks, then they are away.” Respondents described following well-established routines with the aim of maintaining the freshness of food and water and the cleanliness of feeders and drinkers. Care was taken to ensure familiarity not only with the diet but also with the feeders and drinkers themselves. There were also individual mentions of using acidified water to reduce the pH of the gut; and providing “snug areas” in larger yards. Feed and water intake were closely monitored as an indicator of piglet health. Additional indicators of piglet health referred to by respondents included: (i) the dynamics of the group, sociable with an absence of vices and a comfortable level of noise; (ii) the demeanour of individuals, active, bright and curious; and (iii) faecal consistency, clean pigs and pens, no signs of looseness or diarrhoea. The stockperson was described as having a vital role to play in piecing this information together, as one respondent remarked “…they’ll know straight away if you have pigs going back.”

In response to looseness or scouring in pigs in the 2 weeks after weaning, treatment would depend on the severity as judged by the stockkeeper. Typically, affected animals would be managed, for example with the addition of electrolytes to water and supplementary heat. For increased incidents of looseness/scouring, there were mentions of liaising with the farm’s veterinarian and submitting faecal samples for laboratory analysis, and while acknowledging that treatment with antibiotics may be required, as one respondent observed, “zinc is one thing, but antibiotics are something else and it’s easiest not to use any at all.” The need to make staff aware was also emphasised, to prevent movement between affected and non-affected pens and to reinforce additional foot-bathing precautions.

### Quantitative survey: epidemiological considerations

A total of 14 respondents completed the anonymous, online survey. Frequency of the responses for questions on farm and herd characteristics, pig husbandry, management, and health are summarised in Table 1.

**Table 1:** Frequency distribution of investigated farm level parameters (*N* = 14 responses).

Variable	*n*	%
Farm set up		
Indoor system	10	71.40
Outdoor/indoor system	3	21.40
Other	1	7.10
Herd production type		
Breeder through to Finisher	12	85.70
Nursery	2	14.30
Water source used to water pigs		
Mains water supply	4	28.60
Mains water supply, private spring source	2	14.30
Private spring source	6	42.90
Other	2	14.30
Type of ventilation system		
Natural	6	42.90
Natural plus fan	4	28.60
Natural plus fan, automatically controlled natural ventilation	2	14.30
Natural, automatically controlled natural ventilation	1	7.10
Natural, natural plus fan, automatically controlled natural ventilation	1	7.10
Type of pen environment enrichment used		
Wood, ropes	4	28.60
Straw	3	21.40
Wood, other	3	21.40
Wood, ropes, other	2	14.30
Straw, cardboard, wood	1	7.10
Straw, wood	1	7.10

#### Farm and herd characteristics

Most respondents operated an Indoor production system (10/14, 71%), although 3/14 (21%) had Outdoor and Indoor systems (Table 1), consistent with the structure of the United Kingdom pig herd (29). The most reported type of production was Breeder to Finisher (12/14, 86%) although 2/14 (14%) were Nursery Units. Water was variously sourced from private springs (6/14, 43%) and/or mains supply (4/14, 29%).

#### Sow management

Almost half of the respondents (5/12, 42%) reported vaccinating sows against *E. coli* infection, erysipelas disease, and porcine parvovirus (Supplementary Table 1). In the farrowing house, fully slatted plastic slats were the most frequently reported floor type (6/12, 50%). Most respondents reported that farrowing pens were washed and disinfected (11/12, 92%) with an average empty time, where stated, of ≥4 days (7/12, 58%) and 1–3 days (5/12, 42%). Note, herds described as “nursery” (2/14) were excluded in calculations regarding sow and pre-weaned pig management.

#### Pre-weaned pigs

**Figure 3:**
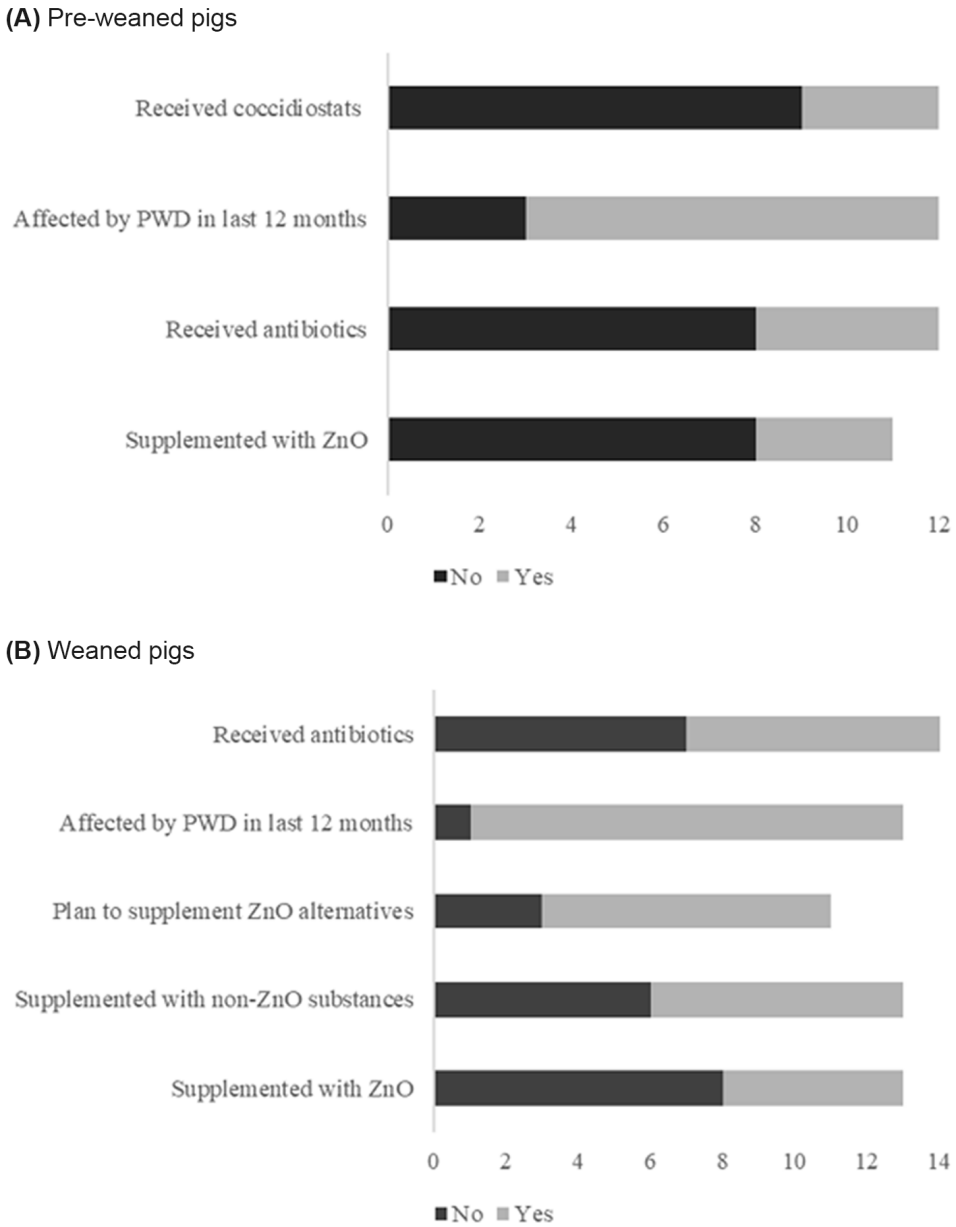
**(A)** Frequency of responses to questions on supplementation with zinc, use of antibiotics and coccidiostats, and experience with diarrhoea in last 12 months in pre-weaned pigs (*n* = 12 responses). *n* = 11 for some variables because the option “do not know” was excluded in calculations shown in figure. **(B)** Frequency of responses on use of antibiotics, experience with post-weaning diarrhoea (PWD) in last 12 months, and supplementation with zinc, non-ZnO substances, or alternatives in weaned pigs. *n* = 11 and *n* = 13 for some variables because the option “do not know” was excluded in calculations shown in figure.

In terms of the health of pre-weaned pigs, most respondents (9/12, 75%) reported that their pre-weaning/suckling piglets had experienced diarrhoea in the last 12 months, and that pre-weaned piglets or weaned pigs did not receive coccidiostats to reduce diarrhoea (Figure 3A, Supplementary Table 2).

Respondents were asked about the type of vaccines pre-weaned piglets receive and just under half of respondents (5/14, 42%) reported vaccinating pre-weaned piglets against porcine circovirus. At the time of completing the survey, most respondents (8/12, 67%) were not supplementing pre-weaned piglets with ZnO in creep (or other) feed before weaning although 3/12 (25%) did so. Most respondents (8/12, 67%) reported that pre-weaned piglets did not receive antibiotics (in feed or water) before weaning, and 4/12 (42%) reported providing antibiotics (in feed or water) before weaning. The most widely reported drinkers were nipple (7/12, 58%) and cup (or bowl drinker) (3/12, 25%). All respondents provided creep feed.

#### Weaning

The typical age of piglets at weaning was 28 days or below (8/14, 57%) followed by 29–35 days (5/14, 36%) and greater than 35 days (1/14, 7%) (Supplementary Table 3). More respondents reported that weaned groups were separated and housed in batches by age (4/14, 29%), size (3/14, 21%), and age and size (3/14, 21%). The vaccines given on the day of weaning or in 7 days immediately afterwards included *Mycoplasma* (3/14, 21%) and *Salmonella* (1/14, 7%). Four respondents (4/14, 29%) offered no vaccine on day of weaning or 7 days post-weaning.

#### Post-weaning

The most reported building type used for weaned pigs was indoor open plan (10/14, 71%), followed by outdoor shelter and field, indoor open plan with internal divisions between pens (2/14, 14%), indoor open plan (with no internal divisions between pens) (1/14, 7%), indoor open plan with internal divisions between pens, “Trowbridge style” (1/14, 7%) (Supplementary Table 3). In the pens of weaned pigs, fully slatted plastic slats were the most reported floor type (10/14, 71%). Half of respondents did not use bedding (e.g., straw) in pens of weaned pigs (7/14, 50%) and 5/14 (36%) used bedding. All respondents reported that pens of weaned pigs were washed and disinfected. The most reported average empty time of the pens of weaned pigs was 1–3 days (10/14, 71%).

Almost all respondents reported weaned piglets affected by PWD in the last 12 months (12/14, 85%) and among the weaned piglets affected by PWD, half of the cases (7/12) were observed 5–10 days after weaning (Table 2, Figure 3B). Some respondents reported a perceived seasonal variation, but most (8/14, 57%) indicated that “*they did not know*”. Some respiratory disease or condition (4/14, 29%) was observed in weaned pigs in the past 12 months. In the 12 months before the survey, 43% (6/14) of respondents reported between 1–5% increase in mortality, and 29% (4/12) of respondents reported a higher 6–10% increase in mortality during the same period.

**Table 2:** Frequency distribution of investigated parameters in weaned pigs (*N* = 14 responses).

Variable	*n*	%
Weaned piglets affected by PWD in last 12 months		
No	1	7.10
Yes	12	85.70
Do not know	1	7.10
When most cases of PWD are observed		
0–4 days after weaning	3	21.40
5–10 days after weaning	7	50.00
11–14 days after weaning	2	14.30
Time of year most cases of PWD are seen		
Autumn (September–November)	3	21.40
Summer (June–August)	1	7.10
Do not know	8	57.10
Other disease (excluding PWD) in weaned pigs in the past 12 months		
Coccidiosis	1	7.10
Respiratory disease or condition	4	28.60
Respiratory disease or condition, Other	1	7.10
Other	2	14.30
None	6	42.90
Mortality in weaned pigs in the past 12 months		
1–5% increase in mortality	4	28.60
6–10% increase in mortality	6	42.90
More than 10% increase in mortality	1	7.10
No change	3	21.40

Most respondents reported that weaned pigs were not fed a diet containing ZnO during the post-weaning period (8/14, 57%) whereas 5/14 (36%) reported feeding a diet containing ZnO during the post-weaning period (Figure 3B, Supplementary Table 4).

Response to an open-ended question elicited comments about the impacts experienced or anticipated from the forthcoming withdrawal of ZnO from weaner diets on herd health. Two long-term non-users of ZnO anticipated little impact. Two respondents that had more recent experience of withdrawing ZnO from diets reported contrasting experiences. One noted that after some transitory impacts in the herd (softer faeces and dirtier weaners) performance was “back to almost normal” while the other reported increased mortality and increased use of antibiotics. The experience of the latter was echoed by another four respondents variously trialling ZnO-free feeding with reports of pigs not thriving (*n* = 3), increased mortality rates (*n* = 3), increased reliance on antibiotics (*n* = 2) and increased demands on the workforce (*n* = 1). Two respondents with nursery units reported experiencing reduced performance and increased mortality in their trials of ZnO-free feeding; one also mentioned increased use of antibiotics and increased demands on staff.

Half of respondents (7/14, 50%) supplemented weaner diets with substances other than ZnO (including probiotics, organic acids, plant extracts or a “premium” type of weaner diet), while 6/14 (43%) did not. Most respondents (8/14, 57%) indicated they would supplement diets with an alternative product to ZnO before and after weaning following the withdrawal of ZnO, while 3/14 (21%) would not.

Half (7/14, 50%) of respondents reported that weaned pigs received antibiotic therapy in-feed/in-water (e.g., as a group) and 7/14 (50%) reported that their weaned pigs did not receive antibiotic therapy in-feed/in-water (e.g., as a group). Most respondents (12/14, 86%) reported that weaned pigs did not receive any other veterinary medicinal product (other than antibiotics) (e.g., as a group).

#### Exploring potential associations between farm practices and PWD

The Fisher exact test was used to examine for association between reported PWD and the following farm practices: fed a diet containing ZnO during the post weaning period, supplemented weaner diets with substances other than ZnO, supplement weaner diets with an alternative product to ZnO before and after weaning following the withdrawal of ZnO from weaner diets, and weaned pigs received antibiotic therapy in-feed/in-water (Supplementary Table 5). Meaningful comparisons could not be made in part due to the observed small sample in the quantitative survey as shown in the 2 × 2 tables (Supplementary Table 5), as such no significant difference was observed between the investigated parameters. The small sample size therefore limited the opportunity for further analyses to explore the associations between PWD and putative husbandry and management factors.

## Discussion

There were contrasts and similarities between the findings from the qualitative enquiries carried out to investigate attitudes among pig producers as the United Kingdom sector prepared for the withdrawal of ZnO and a quantitative survey conducted to identify management and husbandry factors associated with PWD. During the qualitative interviews respondents believed that PWD was being well-controlled in their herds. In contrast almost all respondents to the quantitative survey reported PWD in weaner piglets in the last 12 months, regardless of whether they fed a diet containing ZnO during the post-weaning period or supplemented weaner diets with substances other than ZnO. Although further analyses to explore the associations between PWD and putative factors were not conducted in the presented study due to the small sample size, it is possible that variation in husbandry and management such as dietary practices across farms may have influenced outcomes related to PWD and responses to ZnO withdrawal. It is also possible that if respondents to the quantitative survey were motivated to participate due to problems controlling PWD in their herds this may result in a sample biased towards herds with ongoing problems.

In the qualitative interviews, most respondents agreed with the statement ‘the forthcoming withdrawal of ZnO risks triggering an increase in the use of antimicrobials across the sector’ and indeed we saw half of respondents to the quantitative survey reporting use of antibiotic therapy in-feed/in-water in weaned pigs. The observed increased use of antibiotics echoes experience of producers in Denmark in relation to ZnO withdrawal (22).

Other similarities between qualitative interviews and the quantitative survey were observed in the findings on routine farm practices such as vaccination. For example, all the qualitative interview participants vaccinated piglets, and almost all respondents to the quantitative survey reported vaccinating pre-weaned piglets. In the quantitative survey, although respondents reported vaccinating pre-weaned pigs, most vaccinated against Porcine circovirus (*n* = 5), *Mycoplasma* (*n* = 2) and other (*n* = 5), and only one respondent under the “other” category reported vaccination against a gut pathogen, *E. coli*. However, for sows, most respondents (*n* = 10) reported vaccinating against gut pathogens including *E. coli*, clostridial disease, erysipelas disease, and porcine parvovirus. The potential benefits of using vaccines targeting specific gut pathogens associated with signs of PWD (such as *E coli* and *Salmonella*) in pre-weaned and weaned pigs (30, 31), and sows (32) are documented. However, the extent of awareness and use of such vaccines in pre-weaned and weaned pigs in light of the competing cost of vaccination compared to cost of ZnO, was not clear during the pre-and post ZnO ban and was not investigated in the present study. In sows, the passive immunity generated by vaccines wanes with age and is most useful early on, but less so later at post weaning stage.

**Figure 4:**
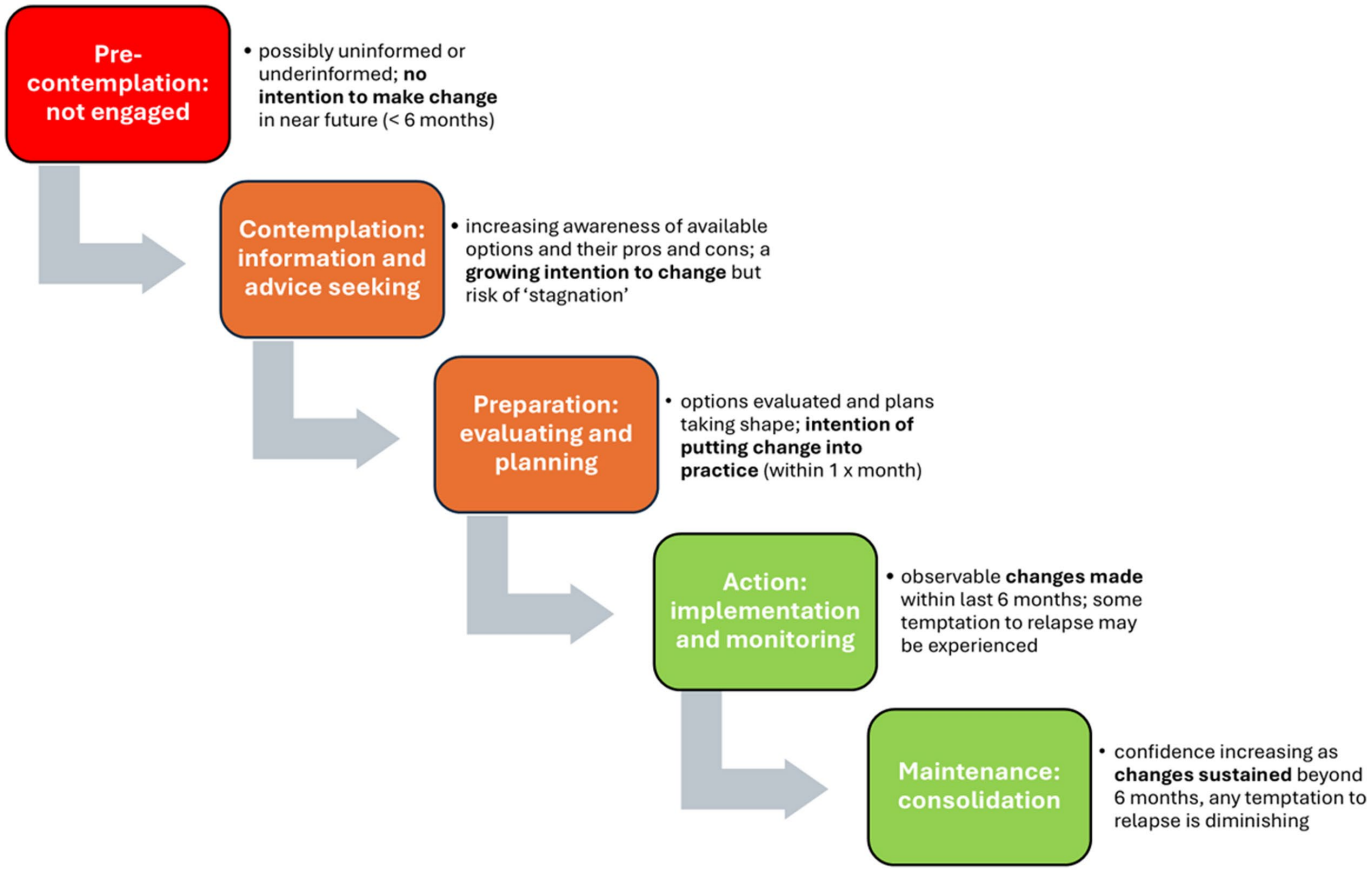
Five stages of behavioural change as described in the transtheoretical model (adapted from Prochaska and Velicer (23); and Doran et al. (26)).

Through the lens of the transtheoretical model (TTM), the processes of change are understood, as illustrated in Figure 4, below, as dynamic (26) and while sometimes linear, at other times cyclical and occasionally relapsing (33). Despite the impending withdrawal date, some producers still felt more information was needed. Although sometimes associated with a resistance to change, pre-contemplation may instead reflect concerns about being insufficiently informed (27). In the context of the United Kingdom’s highly integrated pig sector (34), the impacts of change at any one stage of production, including post-weaning, may extend beyond the farms themselves to affect the wider system, risking misalignments between any two or more of regulatory, market, technology and problem streams (35). For some producers, the volume of ZnO fed to weaners in the United Kingdom was not seen as sufficient to justify its withdrawal in response to concerns about the environmental accumulation of zinc. Elsewhere, a similar disconnect was observed (36) among forest managers, concerned about the environment but not always making connections between environmental impacts and actions undertaken on their land. Indeed, while farmers have been described as “natural adaptors” when it comes to protecting their farms, this does not always extend to a more collective commitment to make change for the common good (37).

In moving from pre-contemplation to contemplation, individuals are said to be shifting from having “no intention to change” to “an intention to change” subject to consideration of associated costs and benefits (38). This shift may, however, be accompanied by “considerable ambivalence” (27) and the value at this early stage of “helping relationships”—where this describes accessing and accepting the support of others in evaluating options—has increasingly come to be recognised (36). In the interviews, respondents associated weaning with an attention to detail and a reliance on well-established routines. While these routines were tailored to the specifics of different units, their common aim was to set animals up for “lifetime performance.” Producers using ZnO either routinely or as a “safety-net” in the event of problems, valued its ease of use, affordability, and effectiveness. Its withdrawal prompted concerns about the costs or consequences for the health and performance of their herds. In evaluating options in response to the withdrawal of ZnO, respondents valued advice from their veterinarian and nutritionist as well as wider farmer networks through discussion groups and online forums. In a context of complex change, the supporting role of these groups is vital (39).

As the decisional balance tilts in favour of change (27) and plans take shape (40) so producers may be described as moving into the preparation stage. This weighing-up of pros and cons takes place in the context of wider drivers and, consistent with Bradford et al. (41), participants in both interviews and the survey saw some contradiction between the forthcoming withdrawal of ZnO as a prophylactic treatment and a sectoral drive to reduce antimicrobial usage (AMU). It has been argued that the greater the confidence in existing practices, the less likely farmers are to plan on changing (37) and responses to the interviews reflected a confidence in the ability of existing, well-established routines to control PWD. Moreover, in the absence of a like-for-like alternative, the need for a site-specific approach and concerns about AMU, there was a sense of unease about moving away from tried and tested feeding strategies that included ZnO.

Nevertheless, “action” in the form of observable change was underway. From the interviews, some respondents described “trialling” the withdrawal of ZnO and they were doing so in the knowledge that ZnO was available as a “safety-net.” Their responses highlight the interconnectedness of pig farming systems as they spoke of monitoring growth rates and the ongoing performance of their units to assist them in evaluating options.

From their research among farmers and veterinarians into the withdrawal of ZnO in Denmark, Kongsted and McLoughlin (22) found that “most experiences with phasing out ZnO were positive.” However, it was also acknowledged that weaning without ZnO, or in the absence of ZnO as a safety-net, “could be a big challenge” with some of their interviewees described as having “experienced great challenges.” In the United Kingdom, replies to the quantitative survey suggest that if these challenges are encountered, they may include decreased performance, increased unevenness across batches, increased levels of scour, increased AMU, increased mortality levels and increased demands on staff. In response to these challenges, some respondents reported having ‘relapsed’ to inclusion of ZnO. After June 2024, this option would be closed although some producers were hoping for adjustments to regulation that could, in time, allow the targeted use of ZnO.

Two quantitative survey respondents were identified as long-term non-users of ZnO (5 years or more), in the “maintenance” stage of change. Although neither reported challenges, both acknowledged that producers transitioning to ZnO-free diets would likely be “fearful” of change. Future studies will investigate to what extent their experience represents the “new normal” as producers adjust to ZnO-free feeding.

In the current study, the qualitative interviews and quantitative survey were conducted before and close to the mandated deadline of ZnO withdrawal. With respect to the TTM, we recognise the effect of this timeline on the choices of producers to act. As reflected by contemporary reporting in the industry journal, PigWorld, we see that rather than a linear process, the withdrawal of marketing authorisations for ZnO in the United Kingdom was subject to changing timelines and accompanying uncertainties. In December 2016, emerging advice from the European Medicines Agency (EMA) recommending withdrawal of marketing authorisations for feeding prophylactic ZnO to pigs was met with concern (NPA is “ready to fight” recommended ban on feed use of zinc oxide—Pig World). By July 2017, the United Kingdom confirmed implementation of withdrawal under the maximum five-year “phasing-out” period (VMD confirms maximum five-year phase out period for zinc oxide—Pig World). However, as the June 2022 cut-off approached, it was announced that quantities of ZnO remaining in the supply chain could be used, up to the date of their expiry (Zinc oxide to be available for a year after June 26 marketing authorisation cut-off—Pig World). This gave United Kingdom producers a 2-year grace period, extending the effective withdrawal date to June 2024. Meanwhile, in February 2023, an industry round table continued to argue for “managed and targeted” use of ZnO to be permitted. These changes were, furthermore, taking place against the backdrop of an industry “in crisis” as debated (June 2022) in the House of Lords (Pig farming industry in England—House of Lords Library) and in the wider context of the Covid-19 pandemic and its aftermath.

## Bias and limitations

Participation in both the qualitative and quantitative strands of enquiry was voluntary. Respondents to the former opted-in as part of a longitudinal herd study; respondents to the latter were self-selecting which may bias the responses. Eight out of 29 participants opted in to the qualitative interviews, all herds were committing valuable support to the project by providing access for the purposes of sample collection and associated information, requesting additional input may have been perceived as overly burdensome by some. Paradoxically, feedback from the eight that participated reflected a positive experience. The qualitative interview comprised open and two closed ended questions which may have biased the responses received. In this study, we wished to elicit information from producers while recognising that their time was limited and by offering pre-determined responses for select questions we sought to minimise the burden on the participants.

A relatively low number of respondents was obtained for the wider industry, open, quantitative survey, limiting the opportunity for further analyses to explore the associations between PWD and putative husbandry and management factors and to extrapolate findings to United Kingdom pig herds. While recognising the limitations of a small sample size, we believe this study still makes a meaningful contribution in terms of improving our understanding of producer perceptions of the impacts of the withdrawal of ZnO on the health and welfare of weaning pigs in the time leading to ZnO withdrawal in the United Kingdom.

As observed by Pandolfi et al. (42), the sector’s complex structure makes achieving a representative sample problematic. They argue, however, that even if findings cannot be generalised, analysis of the sample under consideration is valid and potentially of value in informing future studies. In the current study, all herds recruited to the overarching, longitudinal study were indoor, breeder-finishers hence all participants opting-in to the qualitative component were also indoor, breeder-finishers. By opening the process to the industry through the quantitative component, we sought to give voice to all producers across different production systems. Through seeking opportunities to share feedback with the sector, we aimed to demonstrate the value of participating in data collection and hopefully this will encourage producers to enroll in future similar studies. Further studies with larger sample sizes are recommended to allow for robust statistical analyses to identify potential mitigating factors.

## Conclusion

Where ZnO was used, it was valued and producers felt that PWD was being well-controlled in their herds. This was deemed important, as PWD was associated with negative impacts on the health and welfare of stock and wider herd productivity and performance. Changing from supplementing weaner diets with ZnO, to not supplementing weaner diets with ZnO, prompted concerns that PWD would be less effectively controlled. Moreover, there was a disconnect between the sector’s ongoing drive to reduce antimicrobial usage and the withdrawal of ZnO, as the withdrawal of ZnO was anticipated to trigger increased use of other antimicrobials.

Through application of the TTM to integrate the findings of qualitative enquiries and quantitative survey data, we reflect the staged nature of change with producers variously (pre)contemplating, preparing, acting and maintaining change. Some reported having acted, only to relapse, although after June 2024, reverting to inclusion of ZnO at prophylactic levels was no longer an available option. Respondents spoke of the need to evaluate the impacts of the withdrawal of ZnO on their production systems over time and work will be needed to investigate the uptake and effectiveness of alternative strategies.

## Data Availability

The original contributions presented in the study are included in the article/Supplementary material, further inquiries can be directed to the corresponding author.
