# Supplementary material for: Producer perceptions on the impacts of the withdrawal of zinc oxide on the health and welfare of weaned pigs
Source: Front Vet Sci. 2026 Apr 20;13:1717403. doi: 10.3389/fvets.2026.1717403 (PMC13136187; doi:10.3389/fvets.2026.1717403)
Supplement: Supplementary file 1 [file Data_Sheet_2.pdf]

Supplementary Tables

Supplementary Table 1: Frequency distribution of investigated parameters in sows (n=12 responses).

Variable	n	%
**Vaccinations given to sows**		
E. coli disease, clostridial disease, erysipelas disease, porcine parvovirus	2	16.67%
E. coli disease, clostridial disease, erysipelas disease, porcine parvovirus, Other	1	8.33%
E. coli disease, erysipelas disease, porcine parvovirus	5	41.67%
E. coli disease, erysipelas disease, porcine parvovirus, other	1	8.33%
Erysipelas disease, porcine parvovirus	1	8.33%
None	1	8.33%
Other	1	8.33%
**Floor type in farrowing pens**		
Concrete slatted (fully or partly)	2	16.67%
Fully slatted plastic slats	6	50.0%
Other	4	33.33%
**Farrowing pens washed and disinfected**		
Yes	11	91.67%
Not applicable	1	8.33%
**Average empty time of the farrowing pens**		
≥ 4 days	7	58.33%
1–3 days	5	41.67%

Supplementary Table 2: Frequency distribution of investigated parameters in pre-weaner pigs (n=12 responses).

Variable	n	%
**Type of water supply in the pre-weaned pens**		
Cup (or bowl drinker)	3	25%
Nipple	7	58.3%
Nipple, Cup (or bowl drinker)	2	16.67%
**Creep feed the pigs**		
Yes	12	100%
**Pre-weaning piglets experienced diarrhoea in the last 12 months**		
No	3	25%
Yes	9	75%
**Pre-weaned piglets or weaner pigs received coccidiostats**		
No	9	75%
Yes	3	25%
**Vaccines given to pre-weaned piglets**		
Porcine circovirus	5	41.67%
Mycoplasma	2	16.67%
Other	5	41.67%
**Pre-weaned piglets supplemented with ZnO**		
No	8	66.67%
Yes	3	25%
Do not know	1	8.33%
**Pre-weaned piglets receive antibiotics (in feed or water)**		
No	8	66.67%
Yes	4	33.33%

Supplementary Table 3: Frequency distribution of investigated parameters in weaner pigs (N=14 responses).

Variable	n	%
**Average piglet age at weaning**		
28 days or below	8	57.10%
29-35 days	5	35.70%
Greater than 35 days	1	7.10%
**Weaner groups separated and housed in batches**		
Age	4	28.60%
Age, Size	3	21.40%
Age, Size, Sex	1	7.10%
Sex	2	14.30%
Size	3	21.40%
Size, Sex	1	7.10%
**Vaccines given to weaner pigs at weaning or 7 days post-weaning**		
Mycoplasma	3	21.40%
Salmonella	1	7.10%
None	4	28.60%
Other	4	28.60%
Not answered	2	14.30%
**Building type on your farm used for weaner pigs**		
Indoor open plan (with no internal divisions between pens)	1	7.10%
Indoor open plan with internal divisions between pens	10	71.40%
Indoor open plan with internal divisions between pens, 'Trowbridge style'	1	7.10%
Outdoor shelter and field, Indoor open plan with internal divisions between pens	2	14.30%
**Floor type in the pens of weaned pigs**		
Concrete slatted (fully or partly)	1	7.10%
Fully slatted plastic slats	10	71.40%
Solid ground	2	14.30%
Other	1	7.10%
**Bedding (e.g., straw) in pens of weaned pigs**		
No	7	50.00%
Yes	5	35.70%
Not applicable	2	0.143
**Pens of weaned pigs washed and disinfected**		
Yes	14	100.00%
**Average empty time of pens of weaned pigs**		
1–3 days	10	71.40%
≥ 4 days	4	28.60%

Supplementary Table 4: Frequency distribution of investigated parameters in weaner piglets (N=14 responses).

Variable	n	%
**Type of feed used for weaners**		
Commercial (purchased)	10	71.40%
Commercial (purchased) and Home-milled or mixed	1	7.10%
Home-milled or mixed	3	21.40%
**Weaner pigs fed a diet containing ZnO during the post-weaning period**		
No	8	57.10%
Yes	5	35.70%
Do not know	1	7.10%
**Levels of Zinc oxide in feed**		
<150 ppm	2	14.30%
150-1499 ppm	1	7.10%
1500-2499 ppm	1	7.10%
Do not know	1	7.10%
**Supplement weaner diets with substances other than ZnO**		
No	6	42.90%
Yes	7	50.00%
Do not know	1	7.10%
**Plan to supplement weaner diets with alternatives after ZnO withdrawal**		
No	3	21.40%
Yes	8	57.10%
Do not know	3	21.40%
**Weaner pigs received antibiotic therapy in-feed/in-water**		
No	7	50.00%
Yes	7	50.00%
**Weaner pigs received other medicinal product (other than antibiotics)**		
No	12	85.70%
Yes	1	7.10%
Do not know	1	7.10%

Supplementary Table 5: Associations between select farm practices and reported PWD (n=14 responses).

**Variable**	**PWD**			
	No	Yes	Unsure	P value*
Are your weaned pigs fed a diet containing ZnO during the post-weaning period?				
No	1	6	1	1.00
Yes	0	5	0	
Do not know	0	1	0	
Do you supplement weaner diets with substances other than ZnO?				
No	1	5	0	1.00
Yes	0	6	1	
Do not know	0	1	0	
Following the elimination of ZnO from weaner diets, do you plan to supplement weaner diets with an alternative product to ZnO before and after weaning?				
No	1	2	0	0.30
Yes	0	7	1	
Do not know	0	3	0	
Have your weaned pigs received antibiotic therapy in-feed/in-water?				
No	1	5	1	0.46
Yes	0	7	0	
Have your weaned pigs received any other veterinary medicinal product (other than antibiotics) (e.g. as a group)?				
No	1	11	0	1.00
Yes	0	1	0	
Do not know	0	0	1	

**P value* is based Fisher's exact test and excludes rows with 'do not know' and column with 'unsure.' Note, *P values* shown for completeness only because the small values in the cells do not allow for a meaningful analysis.
