# Supplementary material for: Producer perceptions on the impacts of the withdrawal of zinc oxide on the health and welfare of weaned pigs
Source: Front Vet Sci. 2026 Apr 20;13:1717403. doi: 10.3389/fvets.2026.1717403 (PMC13136187; doi:10.3389/fvets.2026.1717403)
Supplement: Supplementary file 2 [file Data_Sheet_1.pdf]

Supplementary File 2

QUESTIONNAIRE

The survey consists of three parts with questions about your farm set-up, on-farm husbandry and management practices, and the health of your animals.
Section A Farm and herd characteristics
Section B Pig husbandry/Management
Section C Pig health

A. FARM AND HERD CHARACTERISTICS

In this section we are interested in understanding the set-up of your farm and pig herd.

1. In your own words, please describe how, if at all, the forthcoming withdrawal of Zinc Oxide (ZnO) is already impacting or is likely to impact on the health of your herd and the activities of your wider unit. [Text box provided]
2.
3. Which option best describes your farm set up?
    a. Outdoor system
    b. Indoor system
    c. Outdoor/Indoor system
    d. Other (describe)
4. Which option best describes your herd production type?
    a. Breeder only
    b. Breeder through to Weaner
    c. Breeder through to Finisher
    d. Nursery
    e. Nursery through to Finisher
    f. Finisher only
    g. Other (describe)
5. What is the average piglet age at weaning (in days)?
    a. 28 days or below
    b. 29-35 days
    c. Greater than 35 days
    d. Other (describe)
    e. Not known
6. Weaner groups are separated and housed in batches by? Select all that apply.
    a. Age
    b. Size
    c. Sex
    d. Other (describe)
7. What is your current herd size for the following pig stages?

Pig stage	Approx number of pigs on farm	Number of pens in use for each pig stage
Sow		
Weaner		
Grower		
Finisher		

Pen and environment

8. Which option best describes the **building type** on your farm used for **weaner pigs**? Tick all that apply.

    a. Outdoor shelter and field
    b. Indoor open plan (with no internal divisions between pens)
    c. Indoor open plan with internal divisions between pens
    d. Indoor kennel only
    e. Indoor kennel plus outside yard
    f. ‘Trowbridge style’
    g. Other (describe)
9. Which option best describes the **floor type in the farrowing pens** on your farm?
    a. Concrete slatted (fully or partly)
    b. Fully slatted plastic slats
    c. Solid ground
    d. Other (describe)
10. Are **farrowing pens** washed and disinfected on your farm?
    a. No
    b. Yes
    c. Not applicable
    d. Do not know
11. If YES, what is the frequency of washing and disinfecting **farrowing pens**?
    a. Washed after every litter
    b. Washed after every two litters
    c. Washed occasionally (e.g., few times a year)
    d. Other (describe)
    e. Not known
12. What is the average empty time of the **farrowing** pens?
    a. ≥ 4 days
    b. 1–3 days
    c. < 1 day
    d. No empty time
    e. Other (describe)
    f. Do not know
13. Which option best describes the **floor type in the pens of weaned pigs**?
    a. Concrete slatted (fully or partly)
    b. Fully slatted plastic slats
    c. Solid ground
    d. Other (describe)
14. Do you use bedding (e.g., straw) in pens of **weaned pigs**?
    a. No
    b. Yes
    c. Not applicable
    d. Do not know
15. Are pens of **weaned pigs** washed and disinfected on your farm?
    a. No
    b. Yes
    c. Not applicable
    d. Do not know
16. If YES, what is the frequency of washing and disinfecting pens of **weaned pigs**?
    a. Washed after every group
    b. Washed after every two groups
    c. Washed occasionally (e.g., few times a year)
    d. Other (describe)
    e. Do not know
17. What is the average empty time of the pens of **weaned pigs** on your farm?

   a. ≥ 4 days
   b. 1–3 days
   c. < 1 day
   d. No empty time
   e. Other (describe)
   f. Do not know
18. Which option best describes the pen environment enrichment used on your farm? Select all that apply.
   a. Straw
   b. Shavings
   c. Cardboard
   d. Wood
   e. Ropes
   f. Other (describe)
19. Which option best describes the type of ventilation system used on your farm? Select all that apply.
   a. Natural
   b. Natural plus fan
   c. Automatically controlled natural ventilation
   d. Other (describe)

B. PIG HUSBANDRY/MANAGEMENT

In this section we are interested in understanding the day-to-day care of pigs on your farm.

20. Do you creep feed the pigs on your farm?
   a. No
   b. Yes
   c. Not applicable
   d. Do not know
21. What type of feed is used for weaners? Select all that apply.
   a. Commercial (purchased)
   b. Home-milled or mixed
   c. Commercial (purchased) and Home-milled or mixed
   d. Do not know
22. What is the water source used to water pigs on your farm? Tick all that apply.
   a. Mains water supply
   b. Private spring source
   c. Other source (describe)
23. Which option best describes the type of water supply in the pre-weaned pens? Select all that apply.
   a. Nipple
   b. Cup (or bowl drinker)
   c. Other (describe)
   d. Not applicable

SOWS

24. Which, if any, vaccinations are given to sows? Tick all that apply.
   a. None
   b. *E. coli* disease
   c. Clostridial disease
   d. Erysipelas disease
   e. Porcine parvovirus
   f. Other (describe)

PRE-WEANED AND WEANED PIGS

25. What kind of vaccines, if any, do your **pre-weaned** piglets receive?
    a. None
    b. E. coli disease
    c. Salmonellosis
    d. Porcine circovirus
    e. Mycoplasma
    f. Other (describe)
26. Are your **pre-weaned piglets** supplemented with Zinc oxide in creep (or other) feed before weaning?
    a. No
    b. Yes
    c. Do not know
27. If YES, which option best describes the levels of Zinc oxide in feed?
    a. 1500-2499 ppm
    b. 150-1499 ppm
    c. <150 ppm
    d. Other (describe)
    e. Do not know
28. Do your **pre-weaned piglets** receive antibiotics (in feed or water) before weaning?
    a. No
    b. Yes
    c. Do not know
29. What kind of vaccines, if any, do your **weaner** pigs receive at weaning or in the 7 days post-weaning?
    a. None
    b. E. coli
    c. Salmonella
    d. Mycoplasma
    e. Other (describe)
30. Are your **weaner pigs** fed a diet containing Zinc Oxide during the post-weaning period?
    a. No
    b. Yes
    c. Do not know
31. If YES, which option best describes the levels of Zinc oxide in feed?
    a. 1500-2499 ppm
    b. 150-1499 ppm
    c. <150 ppm
    d. Other (describe)
    e. Do not know
32. Do you supplement **weaner diets** with substances other than Zinc Oxide? These may include probiotics, organic acids, plant extracts or a "premium'' type of weaner diet.
    a. No
    b. Yes
    c. Do not know
33. Following the elimination of Zinc Oxide from weaner diets, do you plan to supplement **weaner** diets with an alternative product to Zinc Oxide before and after weaning?
    a. No
    b. Yes
    c. Do not know
34. Have your **weaner pigs** received antibiotic therapy in-feed/in-water (e.g. as a group)?
    a. No
    b. Yes

c. Do not know

35. Have your **weaner pigs** received any other veterinary medicinal product (other than antibiotics) (e.g. as a group)?
    a. No
    b. Yes
    c. Do not know
36. Have your **pre-weaned piglets or weaner pigs** received coccidiostats to reduce diarrhoea?
    a. No
    b. Yes
    c. Do not know

C. PIG HEALTH

In this section we are interested in understanding health aspects of your pigs.

PRE-WEANED AND WEANED PIGS

37. Have the **pre-weaning/suckling piglets** on your farm experienced **diarrhoea** in the last 12 months?
    a. No
    b. Yes
    c. Unsure
38. Have the **weaner piglets** on your farm been affected by **post-weaning diarrhoea** in the last 12 months?
    a. No
    b. Yes
    c. Unsure
39. If YES to **post-weaning diarrhoea in weaner piglets**, when do you observe <u>most cases</u> of post-weaning diarrhoea on your farm? Select one.
    a. 0–4 days after weaning
    b. 5–10 days after weaning
    c. 11–14 days after weaning
    d. Other (describe)
    e. Do not know
40. If YES to **post-weaning diarrhoea in weaner pigs**, what time of year do you see <u>most cases</u> of post-weaning diarrhoea?
    a. Spring (March-May)
    b. Summer (June-August)
    c. Autumn (Sep-Nov)
    d. Winter (Dec-Feb)
    e. Do not know
41. In your opinion, what, if any **challenges**, do you experience managing **post-weaning diarrhoea in weaner pigs** on your farm?
42. Have the **weaner pigs** had any other disease (excluding post-weaning diarrhoea) in the past 12 months?
    a. None
    b. Respiratory disease or condition
    c. Coccidiosis
    d. Swine dysentery
    e. Other (describe)
43. Have you observed an increase in deaths/mortalities in **weaner pigs** on your farm in the past 12 months?
    a. No change
    b. 1-5% increase in mortality

c. 6-10% increase in mortality
d. More than 10% increase in mortality
e. Do not know

44. Please use this space to add any additional points that you wish to include about any aspect of the weaning process and the control of post weaning diarrhoea, the use of Zinc Oxide (ZnO), or its forthcoming withdrawal. [Text box provided]

Thank you for your interest and time in contributing to this study.
