# Supplementary material for: Producer perceptions on the impacts of the withdrawal of zinc oxide on the health and welfare of weaned pigs
Source: Front Vet Sci. 2026 Apr 20;13:1717403. doi: 10.3389/fvets.2026.1717403 (PMC13136187; doi:10.3389/fvets.2026.1717403)
Supplement: Supplementary file 3 [file Data_Sheet_3.pdf]

Page 3: Questions about herd health management at weaning

3. Please can you confirm whether or not you were aware of the forthcoming withdrawal of Zinc Oxide (ZnO) before entering this study?

- ○ Yes
- ○ No

3.a. What is your understanding of why ZnO is being withdrawn in the UK and to what extent do you agree with the rationale for its withdrawal?

3.b. Thinking about using ZnO as a feed additive to help prevent Post Weaning Diarrhoea (PWD) in weaning pigs - please use the five-point scale below to indicate to what extent you disagree or agree that ...

Please don't select more than 1 answer(s) per row.

	Strongly disagree	Disagree	Unsure	Agree	Agree strongly
More information is needed about the implications for producers of the forthcoming withdrawal of Zinc Oxide (ZnO)	☐	☐	☐	☐	☐
The forthcoming withdrawal of Zinc Oxide (ZnO) will make it more difficult to prevent and control Post Weaning Diarrhoea (PWD) on my unit	☐	☐	☐	☐	☐
The forthcoming withdrawal of Zinc Oxide (ZnO) risks triggering an increase in the use of antimicrobials across the sector	☐	☐	☐	☐	☐

3.c. In your own words, please describe how, if at all, the forthcoming withdrawal of Zinc Oxide (ZnO) is already impacting or is likely to impact on the health of your herd and the activities of your wider unit…

Questions 4 to 7 ask about the signs you look out for to monitor piglet health in the first two-weeks immediately after weaning; and then in the period beyond that...

4. In your own words, what do you look for in the 2-weeks immediately after weaning to indicate that weaned pigs are thriving (e.g. lively and alert; eating and drinking well...)

5. And still thinking about the 2-weeks immediately after weaning, what do you look out for to indicate that weaned pigs may be struggling (e.g. shivering, scouring...)

6. Beyond those first two-weeks post weaning, what are the indicators that you look out for as signs of weaners progressing well…

7. And still thinking beyond those first two-weeks post weaning, what are the indicators that you look out for as early warning signs or problems among your weaners…

Questions 8 to 11 explore aspects of weaning in more detail; some use a simple scale and some are open for you to respond in your own words…

8. Using a simple 5-point scale, please tell me to what extent you: strongly disagree / disagree / are unsure / agree / or strongly agree to each one of these three statements…

Please don't select more than 1 answer(s) per row.

	Strongly disagree	Disagree	Unsure	Agree	Agree strongly
Weaning is challenging for piglets	☐	☐	☐	☐	☐
Reducing the challenges of weaning helps to reduce the risk of Post Weaning Diarrhoea (PWD)	☐	☐	☐	☐	☐
At present, Post Weaning Diarrhoea (PWD) is being well-controlled in my herd	☐	☐	☐	☐	☐

9. In your own words, please outline the main measures in place on your unit to minimise the risk of Post Weaning Diarrhoea (PWD) in terms of the management of sows, piglets and the wider farm environment...

	Overview of Main Measures
Management of sows	
Pre-weaning management of piglets	
Post-weaning management of piglets	
Management of the wider farm environment	

10. Of the various measures in place on your unit to control PWD that you have outlined in Q9, above, please indicate which two of these you would identify as being the most important and use the drop down boxes to record how easily implemented and affordable you think each one of these is ...

	Main measures	Overall ease of implementation	Overall cost of implementation
Most important measure		Please select	Please select
2nd most important measure		Please select	Please select

11. If you had an outbreak of scours in the two-weeks immediately after weaning, what would you do to treat affected pigs; and to protect unaffected pigs?

The last two questions reflect more widely on the control of PWD.

12. What are your main sources of advice about piglet health at weaning? Please tick all that apply

- ☐ Veterinarian
- ☐ Nutritionist
- ☐ Feed Provider
- ☐ Farm Consultant

- [ ] Discussion Group - face-to-face
- [ ] Discussion Group - online/virtual
- [ ] Other farmers (informal)
- [ ] Trade press
- [ ] OnLine
- [ ] Research Institute
- [ ] Levy Board
- [ ] Friends/family
- [ ] Other

13. Please use this space to add any additional points that you wish to include about any aspect of the weaning process and the control of PWD, the use of Zinc Oxide (ZnO), or its forthcoming withdrawal...

That completes the survey.
